# END-OF-LIFE CARE PROCESSES IN ASSISTED LIVING COMMUNITIES SPECIALIZING IN MEMORY CARE

**DOI:** 10.1093/geroni/igae098.1660

**Published:** 2024-12-31

**Authors:** Emmanuelle Belanger, Nicole Rosendaal, Xiao (Joyce) Wang, Kali Thomas, Joan Teno, Melissa Clark

**Affiliations:** Brown University, Providence, Rhode Island, United States; Brown University, Providence, Rhode Island, United States; Brown University, Providence, Rhode Island, United States; Johns Hopkins University, Baltimore, Maryland, United States; Brown University, Providence, Rhode Island, United States; Brown University, Providence, Rhode Island, United States

## Abstract

Assisted living (AL) is a common site of death. Many AL communities offer specialized memory care (MC), yet we know little about how their end-of-life (EoL) care practices might differ. We conducted a nationally representative survey of 2,084 AL administrators (43% response rate) about EoL care between 2021-2023. Over half offered MC, with 41.7% of AL communities offering both general AL and MC (combined AL), and 9.3% MC only. Combined ALs were larger (44.7% had 100+ beds, compared to 2.3% in MC only and 14.1% in general AL). ALs offering MC were more likely to report having residents who died in the prior 12 months (88.4% combined AL, 90.0% MC only, 68.9% general AL), which is consistent with a majority reporting a policy of retaining residents in need of EoL care (82.9% combined ALs, 84.6% MC only, and 58.1% general AL only). AL communities offering MC were more likely to have staff available 24/7 to administer subcutaneous injections (67.3% combined AL, 71.1% MC only, 55.8% general AL). Similar opportunities to improve hospice care were reported, with only 32.0% of administrators answering “all of the time” when asked if residents referred to hospice were assessed within 24 hours. Important differences in key processes of care exist between AL offering MC and those providing general AL. Future research is needed to examine how these impact the quality of end-of-life care.

